# Evaluating the Hearing‐Related Quality of Life in People With Intellectual Disabilities

**DOI:** 10.1111/jir.70036

**Published:** 2025-09-18

**Authors:** Susanna Zielonkowski, Philipp Mathmann, Awa Naghipour, Susanne Wasmuth, Lukas Prein, Ross Parfitt, Werner Brannath, Martin Scharpenberg, Vincent Jankovic, Anja Neumann, Katharina Schwarze, Karolin Schäfer, Christian Speckemeier, Corinna Gietmann, Katrin Neumann

**Affiliations:** ^1^ Department of Phoniatrics and Pediatric Audiology, University Hospital Muenster University of Muenster Muenster Germany; ^2^ Department of Otorhinolaryngology, Head and Neck Surgery, Johannes Wesling Klinikum Minden Ruhr‐University Bochum Minden Germany; ^3^ South African College of Music University of Cape Town Cape Town South Africa; ^4^ Competence Center for Clinical Trials Bremen University of Bremen Bremen Germany; ^5^ Institute for Health Care Management and Research University of Duisburg‐Essen Essen Germany; ^6^ Institute for Special Needs Education (d/Deaf and Hard of Hearing) University of Duisburg‐Essen Germany; ^7^ Essener Forschungsinstitut fuer Medizinmanagement–EsFoMed GmbH Essen Germany

**Keywords:** assessment, hearing loss, intellectual disability, quality of life

## Abstract

**Background:**

People with intellectual disabilities are more likely to have hearing loss than the general population, but in most cases, it remains unrecognised and unmanaged. The aims of this study were to determine whether the hearing status of people with intellectual disabilities can be correctly evaluated by themselves and/or their caregivers, and whether hearing loss compromises the hearing‐related quality of life of people with intellectual disabilities.

**Method:**

In the prospective cohort study, HörGeist, 1053 individuals with intellectual disabilities received hearing screening and, where necessary, diagnostic assessment and intervention within their living environment. A self‐developed multipart questionnaire, including items regarding hearing‐related quality of life, was answered by caregivers of the participants and was cross‐checked with the results of the hearing tests. A multivariable regression was performed to verify an association between the hearing‐related quality of life score and the degree of hearing loss.

**Results:**

Hearing loss was diagnosed in 463 (44.0%) participants; thereof, only 120 (25.9%) cases were known beforehand. In 404 participants (59.0%) and 580 caregivers (61.5%), hearing status was rated correctly; it was overestimated in 34.6% and 33.4%, respectively (sensitivity: 0.223/0.271, respectively). The mean hearing‐related quality of life score was 3.0 of 4 possible points. The multivariable regression revealed a small but significant association between the degree of hearing loss and hearing‐related quality of life (*β* = −0.069; *p* < 0.001; adjusted *R* ^*2*^ = 0.081).

**Conclusions:**

Regular audiometric tests are recommended for improving the hearing‐related quality of life in people with intellectual disabilities.

## 1 Introduction

Hearing loss (HL) affects approximately 20% of the world's population and up to 1.9% of children (Haile et al. 2021; World Health Organisation 2021). The WHO Burden of Disease Study (2019) reported this as the third most common reason for years lived with disability (Haile et al. 2021). In Germany, the prevalence of HL in adults is 15.7% (von Gablenz and Holube 2015).

People with intellectual disabilities make up approximately 1% of the population (Maulik et al. 2011) and have a significantly higher risk of being affected by HL than the general population (Herer 2012; Meuwese‐Jongejeugd et al. 2006; Willems et al. 2022). One example of this finding is a multicentre study by Willems et al. (2022) on the Special Olympics, a sporting event for people with intellectual disabilities, where 58.7% showed abnormalities in hearing screening, with HL confirmed in 27.0% of cases. HL also occurs earlier in people with intellectual disabilities than in the general population, especially in cases of trisomy 21 (Meuwese‐Jongejeugd et al. 2006).

People with intellectual disabilities suffer from a significant deficiency in hearing care. One reason for this is that HL often goes unrecognised and undiagnosed (Meuwese‐Jongejeugd et al. 2006). For instance, 74%–83% of the hearing disorders identified at the German Special Olympics in 2004, 2006 and 2008 were not previously known and were therefore unmanaged (Hey et al. 2014; Hild et al. 2008; Neumann et al. 2006).

This undersupply of care may be the result of other medical and health issues being more in the foreground, leading to HL being less often perceived and perhaps considered comparatively unimportant (Jure et al. 1991). Somatic illnesses can manifest atypically in people with intellectual disabilities, and overlap with typical behaviours of intellectual disabilities (Brem and Stockmann 2020; van Schrojenstein Lantman‐De Valk and Walsh 2008), so that symptoms of HL may be falsely attributed to the intellectual disabilities and vice versa (Jure et al. 1991). In addition, taking medical history and physical examination are often difficult in people with intellectual disabilities (Bösebeck 2017; Brem and Stockmann 2020). Furthermore, individuals with intellectual disabilities rarely complain about HL (Herer 2012), meaning that their caregivers are often unaware of it (Hey et al. 2014; Neumann et al. 2010). Aulbert (2019) investigated the accuracy with which people with intellectual disabilities can assess their hearing status, reporting that most people's self‐assessment matched their measured hearing status. Nevertheless, 20.4% overestimated their hearing abilities, so they would not receive the necessary care. In addition, 10.2% were aware of their limitations before the tests but still had not had a consultation with a doctor.

Greater involvement of caregivers is therefore important for problem‐oriented diagnosis and care of people with intellectual disabilities (Brem and Stockmann 2020; Meuwese‐Jongejeugd et al. 2005). The authors are not aware of any studies that show whether caregivers of people with intellectual disabilities can correctly evaluate the hearing status of their wards in order to recognise a need for care.

Adequate care of HL is also important for the general population as it often impairs quality of life (QoL) (Chia et al. 2007; Huang et al. 2024; The Whoqol Group 1998; Tseng et al. 2018), for example, due to associations with loneliness, (further) cognitive impairment and dementia (Huang et al. 2021; Loughrey et al. 2018). Furthermore, the likelihood of depression increases in correlation with the severity of the HL (Dillard et al. 2023; Lawrence et al. 2020). Reduced social activities and anxiety are also more common with HL (Monzani et al. 2008). QoL is therefore clearly influenced by communication difficulties and the severity of HL (Dalton et al. 2003; Tseng et al. 2018). This appears particularly important for people with intellectual disabilities, because this population in general has been reported to have a poorer QoL than people without intellectual disabilities (Golubović and Skrbić 2013; Simões and Santos 2016a).

Children with HL frequently have developmental language disorders (DLD) (Schönweiler et al. 1998), which can also negatively impact their QoL (Eadie et al. 2018). HL and intellectual disabilities both result in DLD (Zorowka 2008), and this may contribute to the under‐diagnosis of HL in children with intellectual disabilities. For example, DLD often occurs in the context of otitis media (Nittrouer and Lowenstein 2024). In children with trisomy 21, the prevalence of otitis media is particularly high (Hildmann et al. 2002; Shott et al. 2001) increasing the risk of overshadowing between intellectual disabilities and HL.

The QoL of people with HL can be improved by adequate therapy (Borre et al. 2023; Morita et al. 2024), and people with intellectual disabilities and HL have also been shown to profit from therapy for their HL (Shott et al. 2001; Youm et al. 2013). Nevertheless, it should not be forgotten that Deaf social networks and sign language proficiency can positively influence various aspects of the QoL of deaf people (Gerich and Fellinger 2012; La Grutta et al. 2023; Werngren‐Elgström et al. 2003). However, it remains unclear to what extent HL affects the QoL of people with intellectual disabilities and how (audiological) care may contribute to its improvement.

To answer this question, an exploration of the assessment of hearing‐related QoL of people with intellectual disabilities, both with and without HL, is necessary. While several questionnaires to assess hearing‐related QoL and the benefits of therapy exist (Anderson 1989; Cassarly et al. 2020; Cox and Alexander 1995; Löhler et al. 2010; Ventry and Weinstein 1982), the authors are not aware of them having been used specifically with people with intellectual disabilities.

The objectives of this study within the HörGeist project were to determine the following: 1) the reliability of the evaluations of hearing status by the participants with intellectual disabilities (self‐assessment) and their caregivers (external assessment); 2) the impact HL has on the QoL of people with intellectual disabilities.

It was necessary to assess the actual hearing status of a large cohort of people with intellectual disabilities, including the degree of any HL identified, and to correlate these results with those of an evaluation of hearing‐related QoL in this same cohort in order to answer these questions.

## 2 Methods

### 2.1 Participants

A total of 1053 people with intellectual disabilities took part in the population‐based prospective cohort study, HörGeist. Participants of all ages were recruited from the Rhineland in Germany and underwent hearing assessments (comprised of screening and diagnostic parts) within their living environment between March 2021 and September 2023. A detailed study protocol is provided in Schwarze et al. (2023).

Based on an extensive internet search, over 810 facilities for people with intellectual disabilities found in the German Rhineland region were invited to participate in the study and received detailed study information. The 158 participating facilities (19.5% of those invited) then informed all individuals with intellectual disabilities who met the inclusion criteria, and their parents or legal guardians, and provided us with the contact details of those who agreed to participate in the study. Subsequently, an appointment for the hearing tests was arranged with each facility.

**FIGURE 1:**
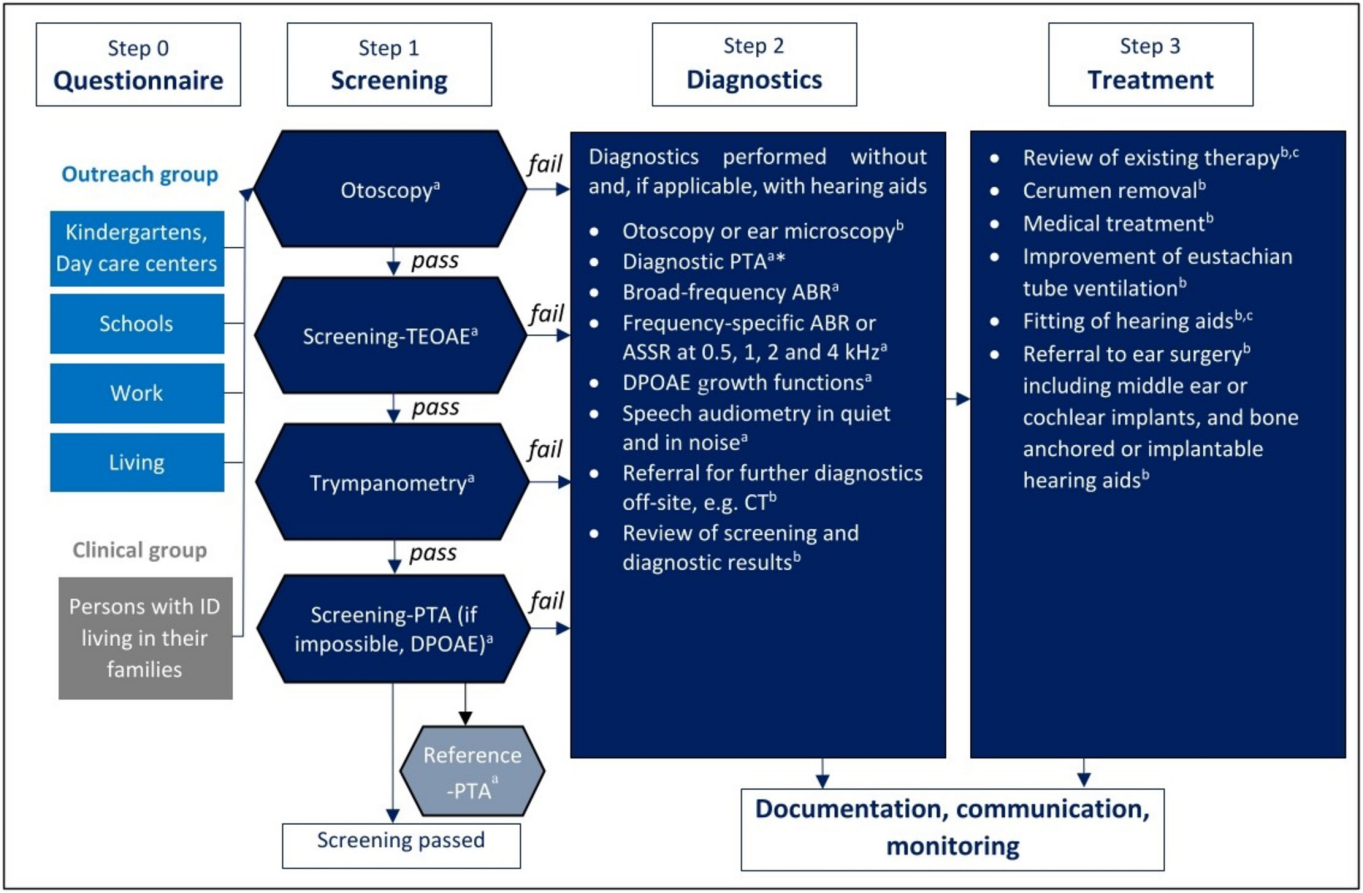
Flow chart of the three‐step HörGeist screening, diagnostic and intervention program (modified according to Schwarze et al. 2023). ^a^Screening staff; ^b^Physician; ^c^Hearing aid acoustician; *In case of failed screening, reference PTA is the diagnostic PTA. ABR, auditory brainstem response; ASSR, auditory steady state response; CT, computed tomography; DPOAE, distortion product otoacoustic emissions; PTA, pure‐tone audiometry; TEOAE, transitory evoked otoacoustic emissions.

The hearing tests were conducted within various facilities for people with intellectual disabilities: nurseries, day‐care centres, schools, living facilities, sheltered workshops, and other workplaces. Participants who did not pass the initial (T0) screening tests immediately had full diagnostic tests and, where necessary, an intervention was initiated or an already existing therapy; for example, use of hearing aids, was monitored. The full procedure is shown in Figure 1. A follow‐up took place 1 year later (T1).

**FIGURE 2:**
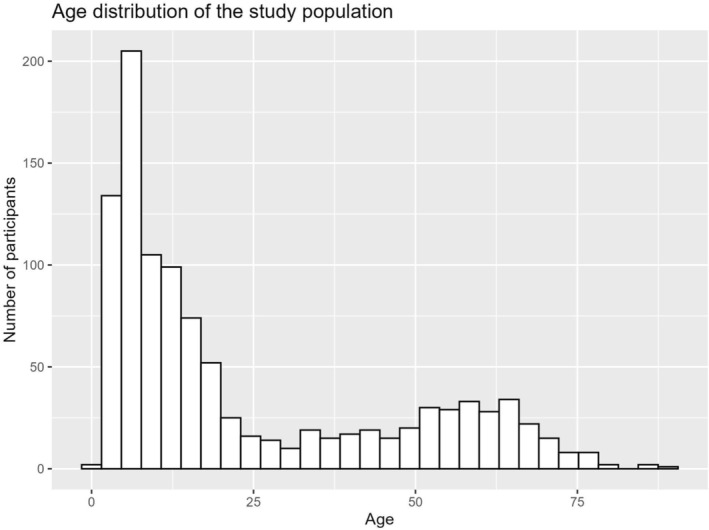
Age distribution of the study population.

Of the 1053 participants, 662 were male (62.9%), 390 female (37.0%) and below 10 intersex (< 1%), corresponding to the distribution in other population‐based studies, which reported a male‐to‐female ratio ranging from 0.7 to 0.9 in adults and from 0.4 to 1.0 in children and adolescents (Maulik et al. 2011). The mean age was 23.7 years (range 1–90 years). Three age groups were distinguished: young children (group K, 0–5 years, *n* = 231), school‐aged children and adolescents (group J, 6–17 years, *n* = 405) and adults (group E, ≥ 18 years, *n* = 417). The detailed age distribution is depicted in Figure 2.

Inclusion criteria were a diagnosed intellectual disability and insurance with the statutory health insurance and project partner, AOK Rhineland/Hamburg. Only individuals for whom undertaking a hearing test posed a risk to themselves or the person conducting the test, e.g., through aggressive or dangerous behaviour, were excluded. This situation occurred in three cases.

After approval of the study by the ethics committee of the Medical Association of Westphalia‐Lippe and the University of Muenster (No. 2020‐843‐f‐S), all participants or their parents or legal guardians received detailed information about the study. This information was provided to participants in plain language when necessary. Written consent to participate was obtained, and additionally, specific caregivers, for example, relatives, legal guardians, caregivers in the facilities or physicians, were released from confidentiality to legitimately answer the questionnaire and provide further necessary information. The study was performed according to the Declaration of Helsinki and the Good Clinical Practice Guidelines (Swart et al. 2015; World Medical Association 2013). The study was funded by the German Innovation Fund (01NVF18038) and is registered in the German Clinical Trials Register (DRKS‐ID: DRKS00024804).

### 2.2 Questionnaire

As part of the project, a multipart questionnaire developed in‐house regarding the hearing‐related medical history, estimated hearing status, concomitant diseases, medication, lifestyle habits and hearing‐related QoL were distributed prior to the first hearing screening (T0). It was administered over the telephone or sent by e‐mail or post to the caregivers of the participants and, in rare cases in which this was not possible, to the participants themselves.

The part of the study presented here focuses on the hearing status of the participants as subjectively estimated by themselves and their caregivers, and on the hearing‐related QoL as judged by the caregivers. The authors are not aware of any instrument with which the hearing‐related QoL of people with intellectual disabilities can be assessed by themselves or others. We, an interdisciplinary expert panel, therefore undertook a multistage process to develop a set of relevant questions based on the scientific literature.

Correlations between the results of the questionnaire and those of the audiometric tests were examined. The 1‐year follow‐up hearing test data (T1) were analysed initially because of their higher reliability; the results of the initial hearing tests (T0) were only used if the T1 data were inconclusive.

### 2.3 Evaluation of Hearing Status

Both the caregivers and the participants themselves were asked to evaluate the participants' hearing status; the caregivers by using the questionnaire and the participants verbally during the initial screening (T0), in plain language or supported by hand signals where necessary (supplementary **eFigure 1**). The responses were summarised in the binary variable “hearing status evaluation” (normal/suspected HL), with the two better and the two worse answers grouped together. This evaluation was then compared with the results of the audiometric tests using cross‐tabulations. Agreement was analysed using Cohen's κ and its 95% two‐sided confidence interval.

### 2.4 Hearing‐Related QoL

Nine features were defined to determine the hearing‐related QoL. These features target aspects of communication (items 2–6, 9a) and behaviour (items 7, 8, 9b, 9c), taking into account the following three QoL factors: social participation, independence and well‐being (Schalock et al. 2008). During item development, particular attention was paid to the relationship of these factors to HL. As outlined in the introduction, HL can result in depression, anxiety and loneliness, which can manifest as sadness, irritability, nervousness and avoidance behaviour (Dillard et al. 2023; Huang et al. 2010; Monzani et al. 2008; World Health Organisation 2022). These aspects have therefore been incorporated into the behavioural features. Moreover, language comprehension and production are relevant factors in the context of social participation and independence (Conti‐Ramsden and Durkin 2008; Hatton 1998; de Jong et al. 2024; Morán et al. 2021; Wong et al. 2005). Consequently, these aspects were surveyed as features of communication. Due to the constraints inherent to the project, it was only feasible to evaluate the hearing‐related QoL using a short set of questions.

The features were answered by the caregivers within the questionnaire at T0 (supplementary **eFigure 2**). For each of the features, 1–4 points were available. The feature ‘language production’ consists of items 4–6, which is a special case: the points allocated in questions 5 and 6 were weighted depending on the percentage given in item 4 for verbal and non‐verbal communication in order to also obtain a total of 1–4 points for this feature.

An overall score quantifying hearing‐related QoL was generated based on these features, with this total score ranging from 1 (very poor) to 4 (very good). In order to compare scores across all individuals, even if not all features were answered, the total score was calculated as the mean of all features that had been completed in each case. A minimum of seven features had to be answered to calculate the score.

A descriptive data analysis was performed for the features (means, standard deviation [SD], item‐total correlation, Cronbach's α). A multivariable regression was used to identify any association between the hearing‐related QoL score and the degree of HL as based on the current WHO classification (World Health Organisation 2021). Age, sex, degree of intellectual disability and previous knowledge of an HL were considered possible confounders (Borre et al. 2023; Michel et al. 2009; Nolte et al. 2019; Nota et al. 2007) and were therefore surveyed in the questionnaire. The degree of intellectual disability was assessed by the caregivers. Table 1 provides an overview of the confounders according to age group.

**TABLE 1:** Overview of confounders according to age group.

Variable	Group K^a^ *n* = 231	Group J^b^ *n* = 405	Group E^c^ *n* = 417	Total *N* = 1053
**Age**				
Mean	4	10	47	24
SD	1	3	17	22
Median (IQA)	4 (3, 5)	10 (7, 13)	51 (34, 61)	13 (6, 42)
Range	1, 5	6, 17	18, 90	1, 90
**Sex**				
Male	144 (62%)	278 (69%)	240 (58%)	662 (63%)
Female	86 (37%)	127 (31%)	177 (42%)	390 (37%)
Intersex	<10 (<1%)	<10 (<1%)	<10 (<1%)	<10 (<1%)
**Degree of intellectual disability**				
Mild	67 (29%)	99 (24%)	107 (26%)	273 (26%)
Moderate	89 (39%)	137 (34%)	202 (48%)	428 (41%)
Severe	53 (23%)	151 (37%)	83 (20%)	287 (27%)
Missing	22 (9.5%)	18 (4.4%)	25 (6.0%)	65 (6.2%)
**Previous knowledge of an HL**				
Yes	23 (10.0%)	46 (11%)	78 (19%)	147 (14%)
No	200 (87%)	333 (82%)	323 (77%)	856 (81%)
Missing	8 (3.5%)	26 (6.4%)	16 (3.8%)	50 (4.7%)

^a^ Young children,

^b^ School‐aged children and adolescents,

^c^ Adults.

The statistical analysis of the pseudonymised data was performed using R (Version 4.4.1, 2024‐06‐14 ucrt). Statistical significance was defined as 2‐sided *p* < 0.05. No multiplicity adjustments were performed, as this part of the study was exploratory in nature.

## 3 Results

HL was detected in 463 (44.0%) participants, of which only 120 (25.9%) cases were known beforehand, and 323 (69.8%) cases were previously undiagnosed (missing answers = 20). Of those with previously unknown HL, 9 (2.8%) cases were severe to complete (≥ 65 dB). Hearing status could not be clearly determined in only 35 (3.3%) participants. The dropout rate between T0 and T1 assessments was 81 (7.7%) participants (Mathmann et al. forthcoming; Neumann et al. forthcoming).

The questionnaire was completed in 1038 (98.6%) cases. There were occasional delays in the administration of the questionnaire due to difficulties in reaching the caregivers. The degree of intellectual disability was evaluated by the caregivers as follows: mild in 25.9% (*n* = 273), moderate in 40.6% (*n* = 428) and severe in 27.3% (*n* = 287) (missing answers in 6.2%, *n* = 65).

### 3.1 Evaluation of Hearing Status

The self‐assessment of the participants' hearing status was completed by 692 (65.7%) participants and the external assessment by 972 (92.3%) caregivers. Table 2 shows the results of the evaluations of hearing status compared with actual measured hearing status. Of the participants whose hearing status could be determined, 404 (59.0%) evaluated it correctly (95% CI, 55.3%–62.6%), as did 580 (61.5%) of their caregivers (95% CI, 58.4%–64.6%). The sensitivity of the self‐assessment was 0.223 (95% CI, 0.180–0.273) and the specificity 0.884 (95% CI, 0.848–0.913). The sensitivity of the external assessment was 0.271 (95% CI, 0.231–0.315) and the specificity 0.906 (95% CI, 0.878–0.928). Cohen's κ was 0.114 (95% CI, 0.054–0.175) for the agreement between the self‐assessment and actual hearing status, and 0.186 (95% CI, 0.134–0.237) for the agreement between caregiver assessment and actual hearing status, indicating poor agreement in both cases.

**TABLE 2:** Cross table showing hearing loss diagnosed and the evaluations of hearing status.

	Hearing loss diagnosed		
	No	Yes	Total
**Hearing status evaluation by participants**			
Normal	336 (49.1%)	237 (34.6%)	573 (83.6%)
Suspected hearing loss	44 (6.4%)	68 (9.9%)	112 (16.4%)
**Total**	380 (55.5%)	305 (44.5%)	685 (100%)
Agreement rate: 0.59 (95% CI, 0.553–0.626) Cohen's κ: 0.114 (95% CI, 0.054–0.175)			
**Hearing status evaluation by caregivers**			
Normal	463 (49.1%)	315 (33.4%)	778 (82.5%)
Suspected hearing loss	48 (5.1%)	117 (12.4%)	165 (17.5%)
**Total**	511 (54.2%)	432 (45.8%)	943 (100%)
Agreement rate: 0.615 (95% CI, 0.584–0.646) Cohen's κ: 0.186 (95% CI, 0.134–0.237)			

### 3.2 Hearing‐Related QoL

Information on the hearing‐related QoL of the participants was provided by 980 (93.1%) caregivers, and a hearing‐related QoL score was calculated for 945 participants (89.7% with at least 7 of 9 features completed). The results of the individual features and the hearing‐related QoL score are documented in Table 3. The Cronbach's α of the score was 0.72, which means an acceptable internal consistency. The total mean hearing‐related QoL score was 3.0 of a maximum of 4 points (SD = 0.5).

**TABLE 3:** Results of the hearing‐related quality of life score.

Feature	Item number	Mean (SD)	Item total correlation	Missing answers (%)
Language comprehension in quiet	2	3.5 (0.8)	0.46	87 (8.3%)
Language comprehension in noise	3	3.0 (0.9)	0.54	98 (9.3%)
Language production	4–6^a^	2.6 (1.2)	0.30	152 (14.4%)
Equanimity	7	3.1 (0.8)	0.43	96 (9.1%)
Happiness	8	3.4 (0.6)	0.38	95 (9.0%)
Communication difficulties	9a	2.5 (1.0)	0.40	125 (11.9%)
Internalisation	9b	2.5 (1.1)	0.39	116 (11.0%)
Externalisation	9c	2.8 (1.0)	0.46	116 (11.0%)
Further signs of hearing problems	9d	3.6 (1.0)	0.24	15 (1.4%)
**Hearing‐related QoL score** ^**b**^		**3.0 (0.5)**		**108 (10.3%)**

^a^ Weighting of item 5 and 6 depending on the percentage given in item 4.

^b^ ≥7 features had to be answered to calculate the score.

**FIGURE 3:**
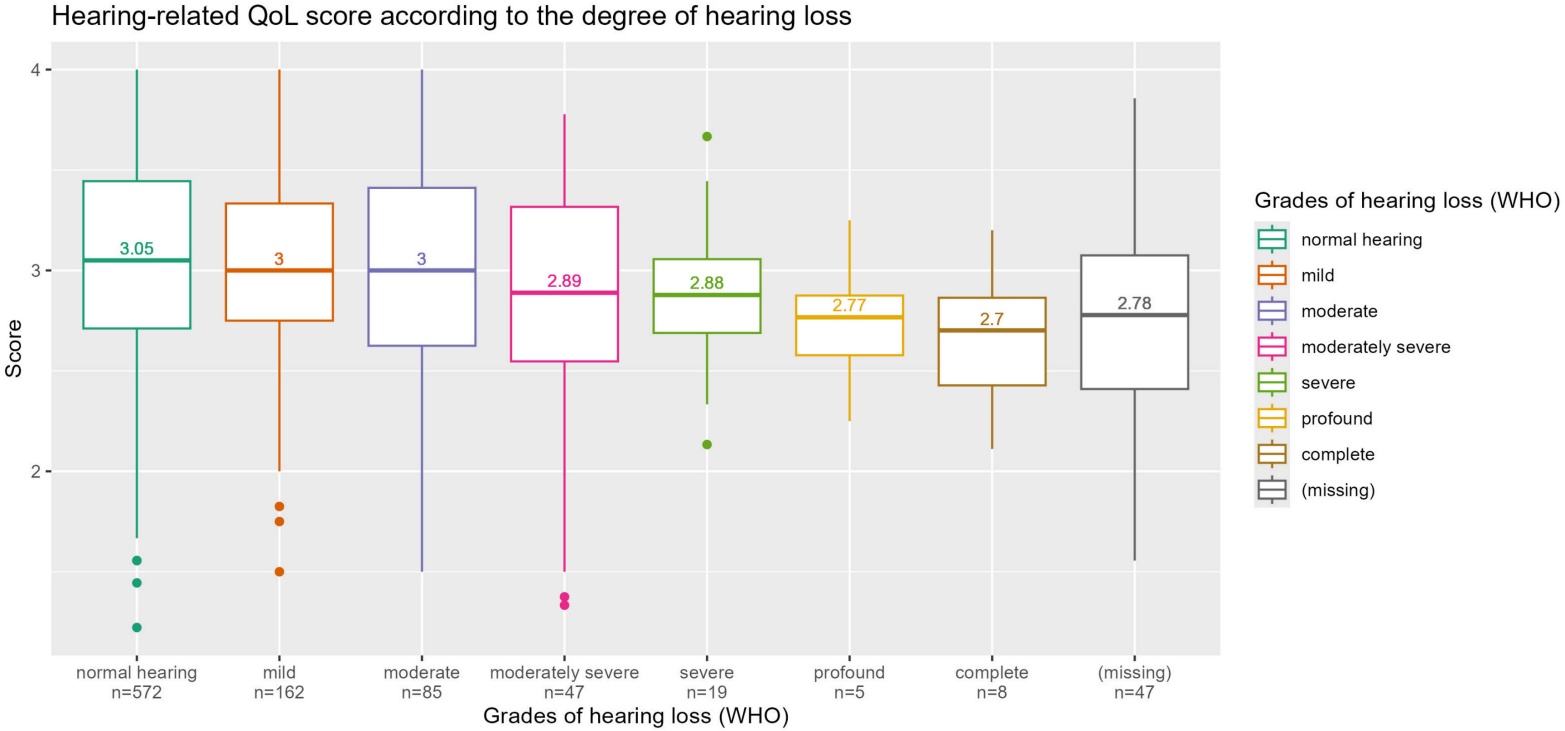
Hearing‐related quality of life score according to the degree of hearing loss.

The hearing‐related QoL scores, segregated according to the degree of HL, are shown in Figure 3. The total mean scores decline slightly with increasing degree of HL, from 3.05 for normal hearing to 2.7 for complete deafness. As depicted in Table 4, the multivariable regression revealed a small but significant association between the degree of HL and the hearing‐related QoL score (regression coefficient β = −0.069; *p* < 0.001; adjusted *R* ^*2*^ = 0.081) when age, sex and the degree of intellectual disability were included as covariates (see regression analysis 1). When also adjusting for previously known HL, the effect was reduced but remained significant (β = −0.038; *p* = 0.04; adjusted *R* ^*2*^ = 0.101) (see regression analysis 2).

**TABLE 4:** Multivariable regression of the hearing‐related quality of life score.

	Regression analysis 1^a^				Regression analysis 2^b^			
	Regression coefficient	Std. error	*t* statistic	*p*	Regression coefficient	Std. error	*t* statistic	*p*
Intercept	3.066	0.039	77.695	<0.001	2.871	0.063	45.682	<0.001
Degree of HL	−0.069	0.017	−4.169	<0.001	−0.038	0.018	−2.098	0.04
Degree of intellectual disability: moderate	−0.137	0.042	−3.245	0.001	−0.152	0.042	−3.589	<0.001
Degree of intellectual disability: severe	−0.262	0.047	−5.546	<0.001	−0.272	0.047	−5.784	<0.001
Age	0.004	0.001	5.046	<0.001	0.004	0.001	4.909	<0.001
Sex: female	0.052	0.036	1.448	0.15	0.062	0.036	1.721	0.09
Prior knowledge of HL: no					0.220	0.054	<0.001	<0.001

^a^ Considered covariates: age, sex, and degree of intellectual disability.

^b^ Considered covariates: age, sex, degree of intellectual disability, and previous knowledge of an HL.

## 4 Discussion

In this part of the larger prospective cohort study, HörGeist, we attempted to answer two questions: 1) How reliable is the evaluation of the hearing status of people with intellectual disabilities by themselves and by their caregivers? 2) To what extent does HL affect the QoL of people with intellectual disabilities?

An unexpectedly large proportion of the participants were diagnosed audiometrically with HL (44.0%). A majority (69.8%) of these cases of HL were previously unknown and therefore inadequately addressed, similar to earlier studies (Hey et al. 2014; Hild et al. 2008; Meuwese‐Jongejeugd et al. 2006; Neumann et al. 2006). One possible reason for this is that people with intellectual disabilities often misjudge their hearing ability, as do their caregivers, probably due to years of habituation.

Only approximately 60% of both respondent groups in our study correctly evaluated the hearing status of the participants, comparable to Aulbert (2019). Of the participants, 34.6% overestimated their hearing ability despite actually having a HL. These people would therefore not seek the necessary help for their HL. This high rate of self‐overestimation, plus the fact that no self‐assessment was feasible in 34.3%, suggests that caregivers should be included in the evaluation of their wards' hearing abilities. However, the caregivers overestimated it almost as often (33.4%) as the participants themselves.

The sensitivity of these subjective evaluations of hearing status is very low, indicating that approximately 75% of all measurable HL goes unrecognised if evaluation is left to the individuals themselves and their caregivers. We strongly recommend that audiometric hearing tests should be performed regularly for all people with intellectual disabilities, in order to detect and provide care for HL at an early stage.

Regular hearing tests are particularly important because our study shows that the degree of HL has a small but significant impact on hearing‐related QoL and that this impact decreases especially when the HL has already been diagnosed. In other words, the mere knowledge of an existing HL already reduces its impact on hearing‐related QoL. Our findings are comparable with studies on people without intellectual disabilities, in which more severe HL also led to poorer overall QoL (Dalton et al. 2003; Ye et al. 2020). The reduction of the influence by prior knowledge about HL seems logical, considering that personal adaptation, communication strategies and coping mechanisms have a relevant influence on hearing‐related QoL (Hallberg et al. 2008; Knutson and Lansing 1990; Warringa et al. 2020) and that these in turn can only be optimised through knowledge about the HL.

The degree of HL was found to have only a minor influence on the hearing‐related QoL and only explained a small amount of the variation in the hearing‐related QoL score. This reflects the results of other studies, which have demonstrated that it takes more than objective audiometric tests to predict hearing difficulties and hearing‐related QoL (Hallberg et al. 2008; Kramer et al. 1996). This may be particularly true for people with intellectual disabilities. Jong et al. (2024), for example, were able to show that other factors, such as speech perception or language proficiency are better suited to predicting psychosocial difficulties in children than the degree of HL. Therapy of HL also has an impact on the QoL (Borre et al. 2023; Morita et al. 2024).

These findings illustrate that in addition to regular hearing tests, the assessment of other constructs, such as language competencies, communication strategies, coping mechanisms and the influence of hearing intervention and rehabilitation, is important to make a targeted contribution to determining and improving the hearing‐related QoL of people with intellectual disabilities. For instance, a communication strategy for individuals with intellectual disabilities and HL could incorporate signs as part of augmentative and alternative communication. This approach has already been utilised in the support of children diagnosed with both intellectual disabilities and developmental language disorders (Neumann et al. 2024). In conclusion, a multifaceted, living environment‐centred approach to care, administered by a multidisciplinary team, warrants consideration (Moeller et al. 2013; Szarkowski et al. 2024).

## 5 Limitations

One limitation of the assessment of hearing‐related QoL in this study is that only the caregivers, but not the participants with intellectual disabilities themselves, were asked about the latter's hearing‐related QoL. Although the involvement of caregivers is an important source of additional information, especially where self‐assessment by people with intellectual disabilities is difficult (Nieuwenhuijse et al. 2024; Simões and Santos 2016b; Verdugo et al. 2005), there is a consensus on including people with intellectual disabilities directly in the assessment of their QoL (Claes et al. 2012; Schmidt et al. 2010; Simões and Santos 2016b; Verdugo et al. 2005; Zimmermann and Endermann 2008). Fellinger et al. (2021), for example, pointed out that a reliable and valid self‐report of QoL can be assessed in people with intellectual disabilities and HL. We assume that the hearing‐related QoL, as judged by the caregivers in our study, does come close to the self‐perception of the participants, based on the fact that associations are often found between the self‐assessment and external assessment of the QoL of people with intellectual disabilities (Claes et al. 2012; Schmidt et al. 2010; Simões and Santos 2016b). There are, however, other studies in which no strong relationship could be proven (Janssen et al. 2005; Scott and Havercamp 2018; Zimmermann and Endermann 2008). Moreover, research findings indicate that individuals with intellectual disabilities generally tend to perceive their QoL positively, often rating it higher than their caregivers (Fellinger et al. 2021; Schmidt et al. 2010; Simões and Santos 2016b; Skotko et al. 2011). Therefore, future studies should aim to assess the hearing‐related QoL directly with people with intellectual disabilities. In light of the brevity and orientational nature of our hearing‐related QoL, we recommend the development and validation of a more detailed questionnaire, potentially building upon the features employed in this study and the work of Schalock et al. (2008) and Clark et al. (2017), among others.

In some exceptional cases, the questionnaire could only be collected after caregivers had received the results of the first hearing tests, due to delays in questionnaire administration. This could have influenced the response behaviour. Nevertheless, it has been shown that the QoL is relatively stable over time regarding temporarily accessible information and more strongly correlated with chronically accessible information (Schimmack and Oishi 2005), so this delay should not have a relevant impact on our data.

## 6 Conclusions

Based on the fact that self‐assessment and external assessment of the hearing ability of people with intellectual disabilities do not provide sufficient reliability for needs‐based care, regular audiometric hearing tests are recommended for all people with intellectual disabilities. This enables timely care and support, especially given that greater severity of HL has an increasingly negative influence on the hearing‐related QoL. The impact of other hearing‐related factors, such as language competencies, communication strategies, coping mechanisms and the influence of a hearing intervention on the hearing‐related QoL of people with intellectual disabilities should be evaluated in order to ensure a focused contribution to improving it.

## Ethics Statement

The study was approved by the ethics committee of the Medical Association of Westphalia‐Lippe and the University of Muenster (No. 2020‐843‐f‐S).

All participants or their parents or legal guardians received detailed information about the study. This information was provided to participants in plain language where possible.

Written consent to participate and a release from confidentiality for caregivers were obtained.

The study was performed according to the Declaration of Helsinki and the Good Clinical Practice Guidelines.

## Conflicts of Interest

The authors declare no conflicts of interest.

## Supporting information


**Figure S1:** Section from the multipart questionnaire to evaluate the hearing status through participants and caregivers (English translation).
**Figure S2:** Section from the multipart questionnaire to assess the hearing‐related quality of life of people with intellectual disability (English translation).

## Data Availability

The participants of this study did not give written consent for their data to be shared publicly, so because of the sensitive nature of the research, supporting data are not available.
