# Supplementary material for: Evaluating the Hearing‐Related Quality of Life in People With Intellectual Disabilities
Source: J Intellect Disabil Res. 2025 Sep 18;69(12):1413–24. doi: 10.1111/jir.70036 (PMC12580484; doi:10.1111/jir.70036)

**Supporting information**

Zielonkowski S, Mathmann P, Naghipour A, et al. Evaluating the hearing-related quality of life in people with intellectual disability.

**eFigure 1.** Section from the multi-part questionnaire to evaluate the hearing status through participants and caregivers (English translation)

**eFigure 2.** Section from the multi-part questionnaire to assess the hearing-related quality of life of people with intellectual disability (English translation)

This supplemental material has been provided by the authors to give readers additional information about their work.

**eFigure 1.** Section from the multi-part questionnaire to evaluate the hearing status through participants and caregivers (English translation)


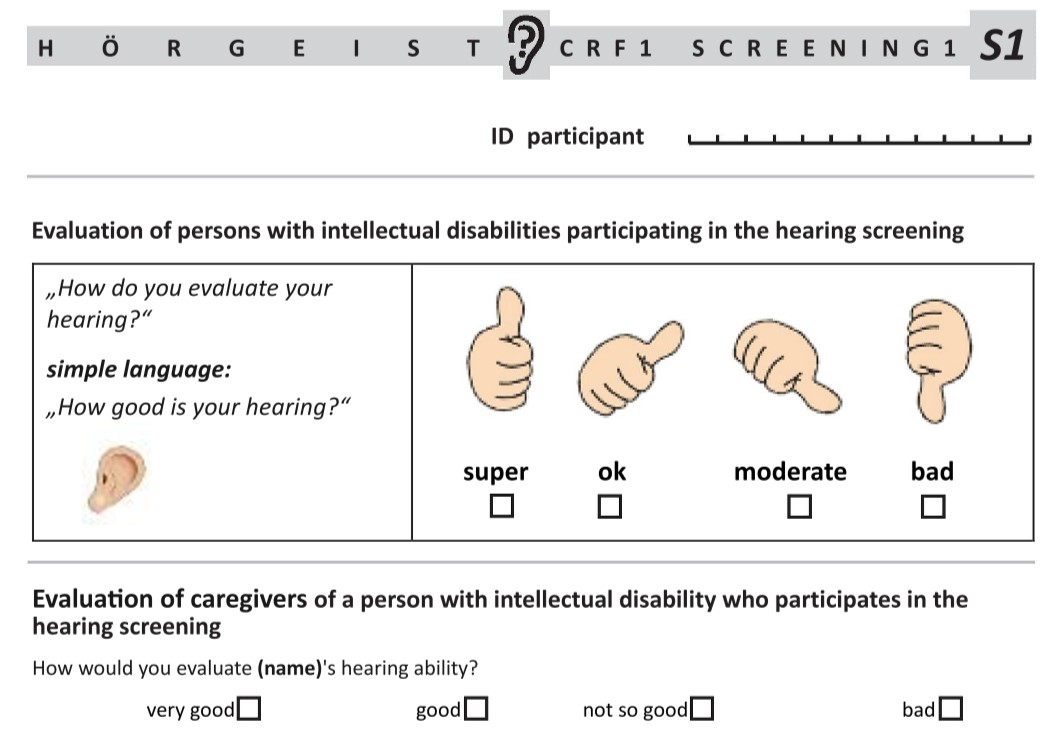


**eFigure 2.** Section from the multi-part questionnaire to assess the hearing-related quality of life of people with intellectual disability (English translation)


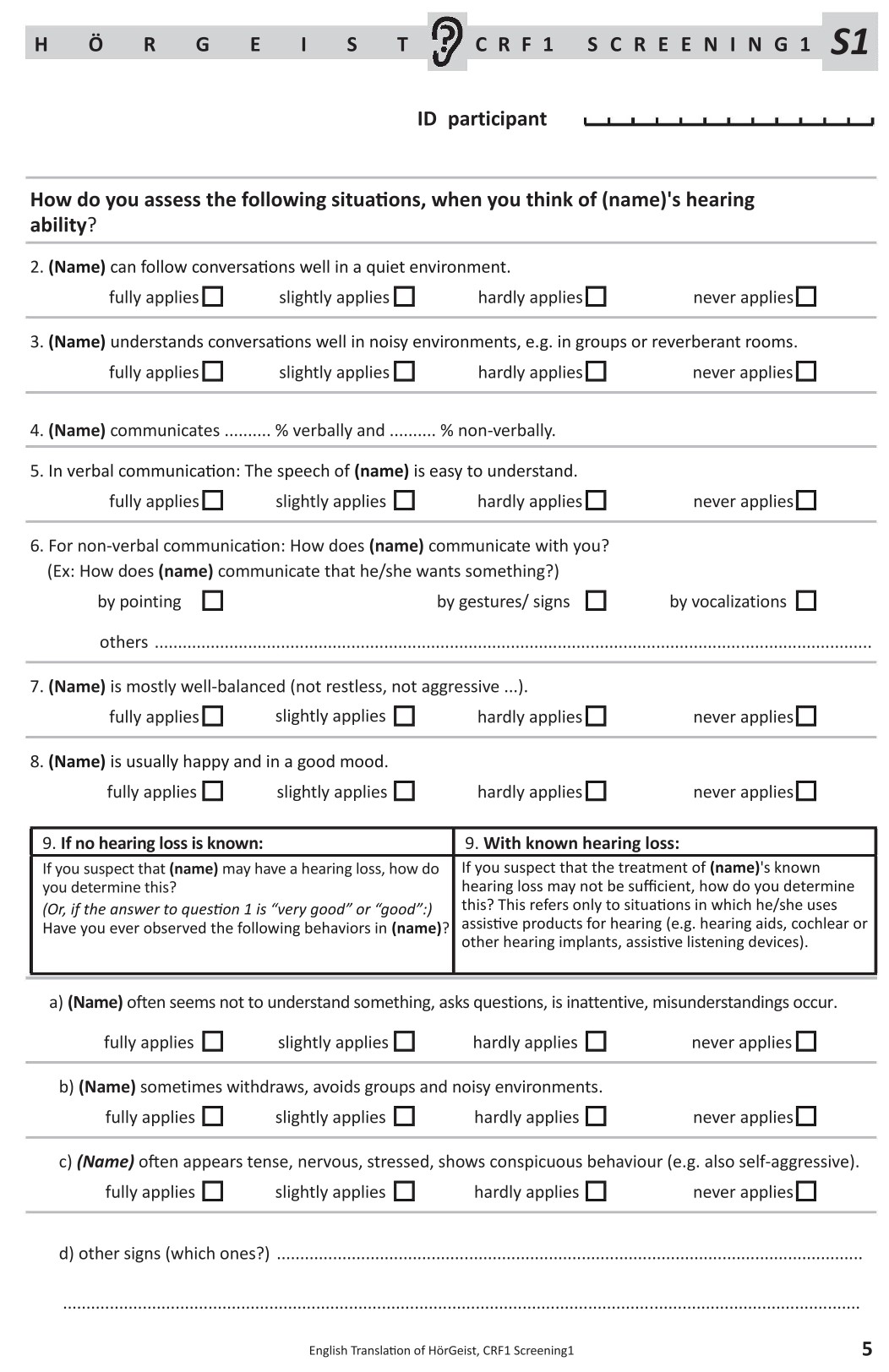

Supplement: Supplementary file 1 — Figure S1: Section from the multipart questionnaire to evaluate the hearing status through participants and caregivers (English translation). Figure S2: Section from the multipart questionnaire to assess the hearing‐related quality of life of people with intellectual disability (English translation). [file JIR-69-1413-s001.docx]
